# Some Association Work

**Published:** 1902-03

**Authors:** 


### EDITORIALS.

### SOME ASSOCIATION WORK.

Hardly a month passes but what we find some association taking a new departure along lines of work that are tending to a stronger and better development of our profession through its more complete organization.

The Manitoba Association recently created prizes for the best papers offered by the new members entering her association ranks, limiting the competition to members who enter during the preceding two years. This is a splendid plan to insure an active interest in association work of the younger members of the profession. The prizes are of useful instruments that should be of double worth to those who receive them, and such a plan might well be followed by many of our State associations.

The publication of all the proceedings of State associations in complete form by one or other of the veterinary periodicals should be a means of stimulating increased interest among the profession of such States so doing, and it will tend to enhance and enlarge the field of journalism by an increased number of readers from whom much encouragement should be received by those who bear the burden of editing and publishing.

The appointment of committees on animal husbandry and press committees in State associations should become very useful bodies in encouraging a closer relation of breeders and veterinarians and of bringing before the public those subjects within our domain that so deeply concern the public welfare.

Recently a local association in Pennsylvania started out in one of its largest cities to determine how far its public institutions sustained by State or municipal aid or under city governmental control furnished to their inmates wholesome and pure milk, and how far they encouraged milk producing on a better scale.

These new lines of work are destined to do much good, and should be encouraged by every member of the profession and extended by every organized association.

